# Phylogenetic and genetic characterization of influenza A H9N2 viruses isolated from backyard poultry in selected farms in Ghana

**DOI:** 10.1002/vms3.809

**Published:** 2022-04-21

**Authors:** Erasmus Nikoi Kotey, Ivy Asantewaa Asante, Mildred Adusei‐Poku, Augustina Arjarquah, Richard Ampadu, David Rodgers, Edward Owusu Nyarko, William Asiedu, Courage Dafeamekpor, Michael R. Wiley, Gifty Mawuli, Richard Asomadu Obeng, Stephen Ofori Nyarko, Vanessa Magnusen, Emmanuel Kodua, Naiki Attram, Shirley Cameron Nimo‐Paintsil, Catherine Pratt, Anne T. Fox, Andrew Letizia, William Kwabena Ampofo

**Affiliations:** ^1^ Noguchi Memorial Institute for Medical Research National Influenza Centre Accra Ghana; ^2^ Department of Medical Microbiology University of Ghana Medical School Accra Ghana; ^3^ U.S. Naval Medical Research Unit No. 3 Ghana Detachment Accra Ghana; ^4^ 37 Military Hospital, Ghana Armed Forces Accra Ghana; ^5^ College of Public Health University of Nebraska Medical Center Omaha Nebraska USA

**Keywords:** backyard poultry, Ghana, H9N2, military

## Abstract

**Introduction:**

Avian influenza viruses (AIV) cause significant economic losses to poultry farmers worldwide. These viruses have the ability to spread rapidly, infect entire poultry flocks, and can pose a threat to human health. The National Influenza Centre (NIC) at the Noguchi Memorial Institute for Medical Research in collaboration with the Ghana Armed forces (GAF) and the U.S. Naval Medical Research Unit No. 3, Ghana Detachment (NAMRU‐3) performs biannual surveillance for influenza viruses among poultry at military barracks throughout Ghana. This study presents poultry surveillance data from the years 2017 to 2019.

**Methodology:**

Tracheal and cloacal swabs from sick and healthy poultry were collected from the backyards of GAF personnel living quarters and transported at 4°C to the NIC. Viral ribonucleic acid (RNA) was isolated and analyzed for the presence of influenza viruses using real‐time polymerase chain reaction (PCR) assays. Viral nucleic acids extracted from influenza A‐positive specimens were sequenced using universal influenza A‐specific primers.

**Results:**

Influenza A H9N2 virus was detected in 11 avian species out of 2000 samples tested. Phylogenetic analysis of viral haemagglutinin (HA) protein confirms the possibility of importation of viruses from North Africa and Burkina Faso. Although the detected viruses possess molecular markers of virulence and mammalian host adaptation, the HA cleavage site anlaysis confirmed low pathogenicity of the viruses.

**Conclusions:**

These findings confirm the ongoing spread of H9 viruses among poultry in Ghana. Poultry farmers need to be vigilant for sick birds and take the appropriate public health steps to limit the spread to other animals and spillover to humans.

## Graphical abstract

Our study confirms the spread of H9 viruses among poultry in Ghana. Poultry farmers need to be vigilant for sick birds and take the appropriate public health steps to limit the spread to other animals and spillover to humans.

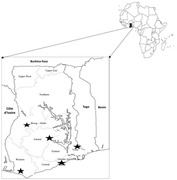

## 1 INTRODUCTION

Influenza is a disease of global public health significance; causing 3–5 million severe illnesses each year resulting in up to 650,000 respiratory deaths in annual seasonal epidemics (Paget et al., 2019). Influenza was reported in Ghana as early as 1918 when the Spanish flu pandemic led to the death of some 100,000 people in a population of 4 million in the then Gold Coast and is described as the worst short‐term demographic disaster in the known history of Ghana (Patterson, 1983). In addition to the global health effects of influenza on international public health systems in Ghana, influenza also remains a high priority respiratory pathogen of U.S. military relevance. Respiratory surveillance activities such as these are essential to better understand the transmission dynamics, epidemiologic trends and emergence of new strains or circulating variants to guide military force health protection (FHP) measures.

Influenza viruses are enveloped and belong to the viral family *Orthomyxoviridae* which are characterized by segmented, single‐stranded, negative sense RNA genomes (Knipe & Howley, 2007). The family consists of seven different genera: influenza viruses A, B, C and D; Isaviruses; Thogotoviruses; and newly proposed Quarjaviruses (made up of Quaranfil, Johnston Atoll and Lake Chad viruses) (Hause et al., 2014; Knipe & Howley, 2007; Presti et al., 2009). Influenza A viruses (IAV) are responsible for seasonal infections (Knipe & Howley, 2007) as well as occasional pandemics in the human population, leading to elevated mortality rates. IAVs are known to infect humans as well as a wide range of animals including pigs, birds, cats, dogs, seals and horses (Horimoto & Kawaoka, 2005; Knipe & Howley, 2007; Mänz et al., 2013). With the exception of H17‐18 and N10‐11, all influenza subtypes have been isolated from wild aquatic birds, which are therefore considered the natural reservoirs of IAVs (Knipe & Howley, 2007). Among the IAV are avian influenza viruses (AIV) such as those belonging to the haemagglutinin (HA) type 5, 7 and 9 groups: these are typically of immense public health concern as they continue to cause significant economic losses to poultry farmers worldwide, due to their ability to spread quickly and infect entire flocks of poultry. AIVs are also a threat to human health including both civilian and millitary populations (Peiris et al., 2007).

In Ghana, troops returning from missions abroad import new breeds of birds that have been identified to be a major source of avian pathogens, including but not limited to AIV. Therefore, The National Influenza Centre, Ghana (NIC) in collaboration with the U.S. Naval Medical Research Unit No. 3 has been conducting routine surveillance for AIV among backyard poultry in military holdings in Ghana. This study reports the characterization of influenza A (H9N2) viruses detected among military backyard/poultry farms in Ghana from 2017 to 2019. The molecular epidemiologic data can further inform preventive health measures and be utilized to develop countermeasures to prevent outbreaks and illnesses that could threaten the operational mission readiness of both the U.S. military and the Ghanaian Armed Forces personnel.

### 1.1 Methodology

Biannual surveillance for AIV included sampling of poultry in all poultry‐keeping units in military barracks across the country. Seemingly healthy bird populations were randomly sampled, whereas morbid birds (such as those presenting with ruffled feathers, cyanosis, watery diarrhoea) were all sampled when encountered. Tracheal and/or cloacal swabs specimens were collected from sampled birds, packaged and shipped on cold‐chain to the NIC for influenza testing. At the NIC, ribonucleic acid (RNA) was extracted from each sample using the QIAamp viral RNA mini kit. Screening for the presence of IAV was conducted and IAV‐positive samples were subsequently subtyped. Master‐mix preparations were performed using the AgPATH (ID) One‐step RT‐PCR kit, with primers and probes, and protocols accessed either from the International Reagents and Resources or from the WHO (WHO, 2017). All PCR testings were performed on the ABI 7500 real‐time PCR device. All gene fragments of the subtype AH9N2 viruses detected were amplified using the universal IAV‐amplifying primers described by Zhou et al. (2009). Briefly, the PCR amplicons, after confirmed on a 1% agarose gel, was quantitated and normalized to about 100 ng using the Qubit fluorimeter. Normalized amplicons were tagmented (fragmented and tagged) using a bead‐link transposome. Tagged nucleic acid fragments were cleaned up by trapping beads with a magnetic stand and performing wash cycles. Subsequently, tagged nucleic acids were PCR amplified using primers linked to index adapters to prepare libraries. Libraries were cleaned up, and aliquots of the libraries were pooled, quantified and set up for sequencing on the Miseq using 2 × 151 base pair paired end sequencing.

### 1.2 Sequence data analysis

Using a pipeline built by U.S. Army Medical Research Institute of Infectious Diseases (USAMRIID), resultant contigs from sequencing were further assembled to generate a consensus sequence for each HA. Consensus sequences were viewed using the BioEdit software v7.2.5, and a nucleotide BLAST on the NCBI platform using these sequences returned aligning sequences, of which the highest identities (> 90%) were downloaded for further alignment with all reference clade‐specific HA sequences. Consensus sequences, BLAST returned sequences and other A H9 sub clades (G1‐like, Y280‐like and Korean‐like viruses) were used to generate a maximum‐likelihood phylogenetic tree applying 1000 bootstrap replicates.

## 2 RESULTS

**FIGURE 1:**
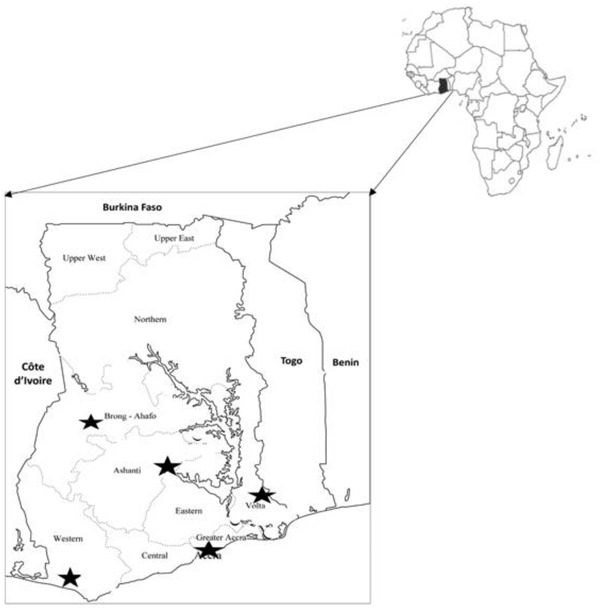
Map of Ghana showing locations of affected poultry farms. Black stars indicate sites in the five regions where tracheal and/or cloacal swabs from poultry tested positive for influenza A (H9N2)

**FIGURE 2:**
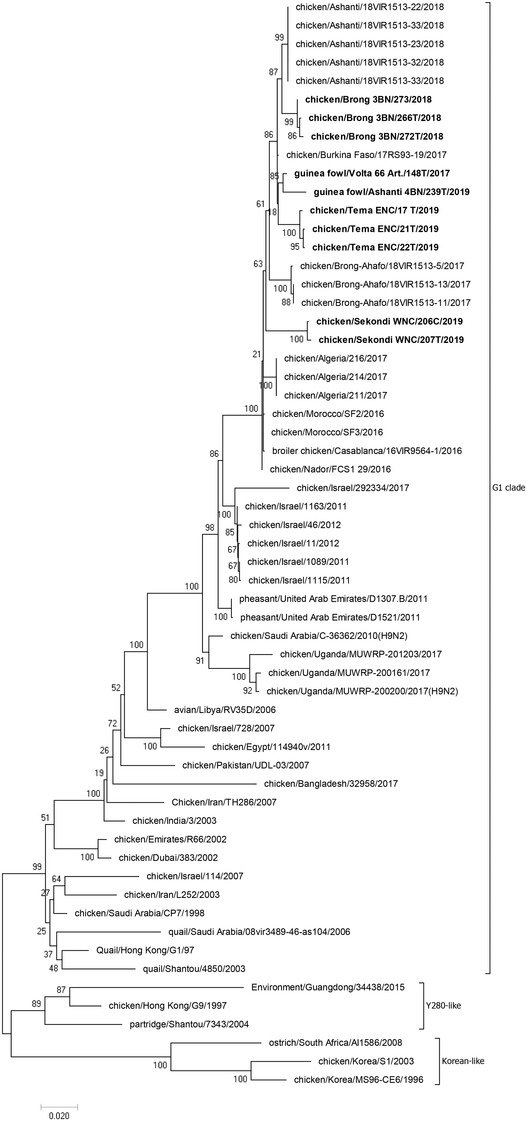
Phylogenetic analysis of influenza A (H9N2) haemagglutinin (HA). Viruses detected in this surveillance are indicated with bold fonts on the phylogenetic tree. This analysis factored several hits after BLAST and the rest of the sequences were clade‐specific reference sequences selected from the GISAID database. Clustering analysis indicates a close relationship among our samples and those earlier reported in Ghana and the strains purported to have originated from the Burkina Faso

Of 2000 birds tested, 11 (comprising five sick and six healthy birds) were confirmed from five of nine sites in five regions sampled by both PCR and sequencing, as the H9N2 subtype of AIV. A map of Ghana highlighting the areas where H9N2 viruses were identified is shown in Figure 1, while details of identified H9N2 viruses and animal source are shown in Table 1. Phylogenetic analyses of HA (Figure 2) showed that H9N2 viruses detected in military installations in Ghana belonged to the G1 clade and were closely related to other H9N2 viruses isolated from Morocco and Burkina Faso. Genetic analyses (Tables 1 and 2) showed that 10/11 viruses possessed the RSSR*GLF cleavage motif on the HA, which is a characteristic of low pathogenic AIV (Nagarajan et al., 2009). One of the 11 viruses had an HA segment that could not be sequenced due to a relatively higher threshold cycle (Ct) value (i.e., Ct > 29).

**TABLE 1:** Description of influenza A (H9N2)‐infected birds, barracks sources, PCR and results of sequencing

					CDC PCR protocols		
Year/ID	Animal	Specimen	Health status	Barracks, region	Type	Subtype	Segment sequenced
17/148	Guinea fowl	Tracheal/cloacal	Healthy	66‐Artillery, Volta	A	H9	1–8
18/266	Chicken	Tracheal/cloacal	Healthy	3rd Infantry Battalion (3 BN), Brong Ahafo	A	H9	1–8
18/272	Chicken	Tracheal/cloacal	Healthy	3rd Infantry Battalion (3 BN), Brong Ahafo	A	H9	1–8
18/273	Chicken	Tracheal/cloacal	Sick	3rd Infantry Battalion (3 BN), Brong Ahafo	A	H9	1–8
19/17	Chicken	Tracheal/cloacal	Sick	Eastern Naval Command (ENC), Tema	A	H9	1–8
19/21	Chicken	Tracheal/cloacal	Sick	Eastern Naval Command (ENC), Tema	A	H9	1–8
19/22	Chicken	Tracheal/cloacal	Healthy	Eastern Naval Command (ENC), Tema	A	H9	1–8
19/206	Chicken	Tracheal/cloacal	Healthy	Western Naval Command (WNC), Sekondi	A	H9	1–8
19/207	Chicken	Tracheal/cloacal	Healthy	Western Naval Command (WNC), Sekondi	A	H9	1–8
19/239	Guinea fowl	Tracheal/cloacal	Healthy	4th Infantry Battalion (4 BN), Ashanti	A	H9	1–8
19/255	Chicken	Tracheal/cloacal	Sick	Central Command Barracks, Ashanti	A	H9	1, 5 and 7

**TABLE 2:** Genetic characterization of influenza A (H9N2) viruses detected in Military barracks

Description	Segment (molecular marker)	Samples										
		2019/17	2019/21	2019/22	2019/206	2019/207	2019/239	2019/255	2018/266	2018/272	2018/273	2017/148
Molecular determinants of virulence	PB2 (147V)	S	O	O	P	O	O	O	O	O	S	S
	PA (127V, 550L, 672L)	P	P	P	P	P	P	N	P	P	P	P
Pathotype based on HA cleavage site motif	HA ((R/KSSR^*^GLF)/ R/KRSR^*^GLF (335–341)	RSSR^*^GLF	RSSR^*^GLF	RSSR^*^GLF	RSSR^*^GLF	RSSR^*^GLF	RSSR^*^GLF	N	RSSR^*^GLF	RSSR^*^GLF	RSSR^*^GLF	RSSR^*^GLF
Molecular determinants of host specificity (adaptation to mammals)	PB2 (318R, 590S, 661T)	P	P	P	P	P	P	P	P	P	P	P
	PB2 (185I)	P	O	O	P	O	O	O	O	O	P	P
	PB1 (13P)	P	P	P	P	P	P	N	P	P	P	P
	PA (100I, 312R, 409N, 291S)	P	P	P	P	P	P	N	P	P	P	P
	^#^HA (155T, 158N, 183N, 226L and 391K)	P	P	P	P	P	P	N	P	P	P	P
	NP (372D)	P	P	P	P	P	P	N	P	P	P	P
	M (15I, 154I, 215A)	P	P	P	P	P	P	P	P	P	P	P
Human‐to‐human transmission markers	PB2 (105)	P	O	P	P	O	P	O	O	O	P	P

*Note*: S = I substitution; O = partially sequenced regions with no base calls; ^*^HA cleavage site: H9 numbering was used to assess all markers, except ^#^HA, which denotes H3 numbering; P = present; and N = segment could not be sequenced.

Multiple markers of mammalian adaptivity, virulence and human‐to‐human transmission were identified in the HA, matrix, polymerase basic 1 (PB1), and polymerase basic 2 (PB2) proteins as well as in the polymerase acidic (PA) segment and the nucleoprotein (NP) (Table 2). The 155T, 158N, 183H, 226L and 391K mutations seen in the HA of 10/11 viruses are required to switch the receptor recognition specificity from avian‐like receptors to the mammalian α2,6‐sialic acid receptors (Kandeil et al., 2014; Li et al., 2020; Wan & Perez, 2007). All the viruses contained mammalian adaptive markers 15I, 154I, 215A and markers 318, 590S and 661T in the matrix and PB2 proteins, respectively, all shown to enhance human‐to‐human transmission (El Houadfi et al., 2016; Kandeil et al., 2014; Zhang et al., 2011). The virulence marker 147V for PB2 was observed for one virus. Also in the PB2, marker 105V responsible for human‐to‐human virus transmission and 185I, a mammalian host‐specific marker, were identified in six and four viruses, respectively (Miotto et al., 2008; Zhang et al., 2011). Mammalian‐specific adaptive motifs on the PB1 segment 13P were identified in 10 of 11 viruses (Wernery et al., 2013). Molecular markers of virulence (127V, 550L and 672L) and mammalian adaptive markers (100I, 312R, 409N and 291S) were detected on the PA segment of the same 10 viruses (Chen et al., 2006; El Houadfi et al., 2016; Kandeil et al., 2014; Rolling et al., 2009). In the NP, 10 viruses contained the 372D mutation, which is required to change the host range from avian to human (Wernery et al., 2013).

## 3 DISCUSSION

Phylogenetic analysis showed that the identified viruses clustered closely with viruses detected earlier in North Africa, supporting the specualtion that these viruses were imported into Ghana from North Africa through Burkina Faso, as posited by Awuni et al. (2019). The borders in the West African subregion are known to be porous, and it is possible that the viruses were introduced through poultry trading between Ghana and Burkina Faso. The presence of the RSSR*GLF cleavage site present in HA of identified AIV confirmed that they belonged to the G1 subclade of the H9 Eurasian lineage. Awuni et al. (2019) showed the presence of H9N2 viruses among poultry in outbreak sites across the Ashanti and Brong Ahafo regions from non‐military commercial farms. In this study however, the H9 viruses were detected among birds sampled from backyard poultry farms in military installations in Volta, Brong Ahafo, Greater Accra, Western and Ashanti regions, where no outbreaks have been reported, providing a firmer indication of the presence of low pathogenic H9 viruses in diverse areas of the country. As posited by Pu et al. (2015), this finding forms a basis that these H9 viruses, although of low pathogenicity, could be gradually disseminating throughout Ghana.

As shown by the genetic analyses, observed single nucleotide polymorphisms associated with mammalian adaptation, virulence and/or enhanced zoonotic transmission are of utmost concern for the likelihood of zoonotic infections. While globally AIV spillover events have so far not demonstrated sustained transmission between human‐to‐human, influenza viruses are constantly evolving, creating the possibility of evolving variants of concern. Military barracks in Ghana, therefore, are of immense interest and concern due to the societal nature of these installations, hence serving as fertile grounds for sustained transmission in the unlikely event of any possible pathogenic zoonotic virus eruption. Nevertheless, during our visit, none of the military farmers exhibited any signs of influenza—like illness. For this reason, samples were not collected from farmers for further investigations.

This study, having documented the presence of AIV H9N2 among both sick and healthy birds populations sampled in Ghanaian military installations, demonstrates the importance of active surveillance in averting potentially massive future outbreaks due to AIV. Further, the identification of transmission and virulence markers warrants continuous surveillance within the country. Subsequently, necessary bioscurity measures such as culling of sick birds should be employed to contain spread. In addition, follow‐up visits must be conducted to also sample and test farmers who tend farms that are confirmed to be affected with AIV as they could be the reservoire for onward transmission of possibly pathogenic strains in the near future. Education on the application of good biosecurity and biosafety practices should also be intensified among Ghanaian farmers, Ghana Armed Forces installations and military partners engaged with these Ghana military sites.

## 4 CONCLUSION

In the wake of numerous outbreaks of avian influenza viruses among poultry in Ghana, we report here the spread of AIV of the H9N2 subtype among backyard poultry in Ghana Armed Forces installations throughout the country. Phylogenetic analysis helps suggest that these viruses may have been introduced into the country from North Africa through Burkina Faso, probably through poultry trade. Isolated viruses harbored several mammalian adaptive motifs. With several avian influenza viruses co‐circulating in Ghana and the West African region and porous borders in the subregion, surveillance systems among flocks in Ghana and other countries should be strengthened to identify sick birds for possible culling and to minimize potential spillover events. With the continuous U.S. DoD presence in Ghana and Ghana Armed Forces military‐to‐military engagements occurring at military installations across Ghana, the incidence and prevalence and genetic characteristics of AIV are a critical FHP concern. Understanding the burden of AIV in circulation at GAF installations provides insight to better inform both U.S. and GAF personnel that may be at risk of zoonotic spillover events and helps base commanders enable countermeasures during military‐to‐military engagements to mitigate the risk of human transmission.

## CONFLICT OF INTEREST

The authors declare no conflict of interest.

## ETHICS STATEMENT

This epidemiologic activity was approved by the Noguchi Memorial Institute for Medical Research Institutional Review Board (IRB) and the Naval Medical Research Center Institutional Review Board and Office of Research Administration as public health surveillance (NAMRU3‐PJT‐21‐01). The Ghana Health Service also concurred that this protocol is exempt from ethical considerations because it falls under public health surveillance and was exempt from both institutional animal care and use committee (IACUC) and IRB evaluation.

## COPYRIGHT/DISCLAIMER STATEMENT

Naiki Attram, Shirley Cameron Nimo‐Paintsil, Anne T. Fox and Andrew Letizia are military Service members or employees of the U.S. Government. This work was prepared as part of their official duties. Title 17, U.S.C., §105 provides that copyright protection under this title is not available for any work of the U.S. Government. Title 17, U.S.C., §101 defines a U.S. Government work as a work prepared by a military Service member or employee of the U.S. Government as part of that person's official duties. The views expressed in this article are those of the authors and do not necessarily reflect the official policy or position of the Department of the Navy, Department of Defense, the U.S. Government nor the institutions affiliated with the authors.

## AUTHOR CONTRIBUTIONS

*Methodology, supervision, writing—original draft and writing—review and editing* : Erasmus Nikoi Kotey. *Supervision, writing—original draft and writing—review and editing*: Ivy Asantewaa Asante. *Writing—review and editing*: Mildred Adusei‐Poku, Courage Dafeamekpor, Richard Ampadu, and David Rodgers*. Supervision and writing—review and editing*: Edward Owusu Nyarko. *Methodology and writing—review and editing*: William Asiedu. *Methodology, writing—original draft and writing—review and editing*: Michael R. Wiley. *Writing—review and editing*: Gifty Mawuli. *Investigation and writing—review and editing*: Richard Asomadu Obeng. *Investigation and writing—review and editing*: Stephen Ofori Nyarko. *Writing—review and editing*: Vanessa Magnusen. *Investigation and writing—review and editing*: Emmanuel Kodua. *Project administration, supervision and writing—review and editing*: Naiki Attram. *Project administration, supervision and writing—review and editing*: Shirley Nimo‐Paintsil. *Writing—review and editing*: Catherine Pratt. *Writing—review and editing*: Anne T. Fox. *Funding acquisition, project administration and writing—review and editing*: Andrew Letizia. *Conceptualization, funding acquisition, methodology, project administration and writing—review and editing*: William Kwabena Ampofo.

### PEER REVIEW

The peer review history for this article is available at https://publons.com/publon/10.1002/vms3.809.

## Data Availability

Data is available from corresponding author upon reasonable request.
